# *Massilia* strains facilitate root growth and reshape root microbiota through diverse salicylic acid hydrolysis pathways

**DOI:** 10.1093/ismejo/wrag082

**Published:** 2026-05-26

**Authors:** Qin Han, Guanghui Zhu, Chenxi Li, Mingbo Li, Chenyijun Guo, Junwen Liao, Jiaming Zhang, Yongliang Wang, Hui Wang, Yuxuan Hong, Ming Li, Ye Zhu, Yuming Gong, Hongsheng Song, Chao Su, Peiwu Li, Xia Li

**Affiliations:** National Key Laboratory of Crop Genetic Improvement, Hubei Hongshan Laboratory, College of Plant Science and Technology, Huazhong Agricultural University, Wuhan, Hubei 430070, China; Key Laboratory of Biology and Genetic Improvement of Oil Crops, Ministry of Agriculture, Oil Crops Research Institute, Chinese Academy of Agricultural Sciences, Wuhan, Hubei 430061, China; National Key Laboratory of Crop Genetic Improvement, Hubei Hongshan Laboratory, College of Plant Science and Technology, Huazhong Agricultural University, Wuhan, Hubei 430070, China; National Key Laboratory for Germplasm Innovation and Utilization of Horticultural Crops, Key Laboratory of Horticultural Plant Biology, Ministry of Education, Key Laboratory of Potato Biology and Biotechnology, Ministry of Agriculture and Rural Affairs, Huazhong Agricultural University, Wuhan, Hubei 430070, China; National Key Laboratory of Crop Genetic Improvement, Hubei Hongshan Laboratory, College of Plant Science and Technology, Huazhong Agricultural University, Wuhan, Hubei 430070, China; Key Laboratory of Biology and Genetic Improvement of Oil Crops, Ministry of Agriculture, Oil Crops Research Institute, Chinese Academy of Agricultural Sciences, Wuhan, Hubei 430061, China; National Key Laboratory of Crop Genetic Improvement, Hubei Hongshan Laboratory, College of Plant Science and Technology, Huazhong Agricultural University, Wuhan, Hubei 430070, China; National Key Laboratory of Crop Genetic Improvement, Hubei Hongshan Laboratory, College of Plant Science and Technology, Huazhong Agricultural University, Wuhan, Hubei 430070, China; National Key Laboratory of Crop Genetic Improvement, Hubei Hongshan Laboratory, College of Plant Science and Technology, Huazhong Agricultural University, Wuhan, Hubei 430070, China; National Key Laboratory of Crop Genetic Improvement, Hubei Hongshan Laboratory, College of Plant Science and Technology, Huazhong Agricultural University, Wuhan, Hubei 430070, China; National Key Laboratory of Crop Genetic Improvement, Hubei Hongshan Laboratory, College of Plant Science and Technology, Huazhong Agricultural University, Wuhan, Hubei 430070, China; Key Laboratory of Biology and Genetic Improvement of Oil Crops, Ministry of Agriculture, Oil Crops Research Institute, Chinese Academy of Agricultural Sciences, Wuhan, Hubei 430061, China; Key Laboratory of Biology and Genetic Improvement of Oil Crops, Ministry of Agriculture, Oil Crops Research Institute, Chinese Academy of Agricultural Sciences, Wuhan, Hubei 430061, China; Key Laboratory of Biology and Genetic Improvement of Oil Crops, Ministry of Agriculture, Oil Crops Research Institute, Chinese Academy of Agricultural Sciences, Wuhan, Hubei 430061, China; National Key Laboratory of Crop Genetic Improvement, Hubei Hongshan Laboratory, College of Plant Science and Technology, Huazhong Agricultural University, Wuhan, Hubei 430070, China; National Key Laboratory of Crop Genetic Improvement, Hubei Hongshan Laboratory, College of Plant Science and Technology, Huazhong Agricultural University, Wuhan, Hubei 430070, China; National Key Laboratory of Crop Genetic Improvement, Hubei Hongshan Laboratory, College of Plant Science and Technology, Huazhong Agricultural University, Wuhan, Hubei 430070, China; Key Laboratory of Biology and Genetic Improvement of Oil Crops, Ministry of Agriculture, Oil Crops Research Institute, Chinese Academy of Agricultural Sciences, Wuhan, Hubei 430061, China; National Key Laboratory of Crop Genetic Improvement, Hubei Hongshan Laboratory, College of Plant Science and Technology, Huazhong Agricultural University, Wuhan, Hubei 430070, China

**Keywords:** *Massilia*, soybean, root growth, SA hydrolysis pathway, root–associated bacterial community

## Abstract

Rhizosphere microbiota play an important role in maintaining plant root growth. However, the physiological and molecular basis of microbial regulation of root traits remains poorly understood. Here, we report that *Massilia* efficiently colonizes roots and promotes root elongation. In particular, the M117 strain of *Massilia* significantly inhibits salicylic acid (SA)-related immune signaling, which is required for M117-mediated root elongation. M117 can directly degrade SA, thereby reducing SA levels in the roots. Integrated omics reveal the presence of multiple SA hydrolytic pathways in M117. Among them, the *NagGHAaAb* pathway is strongly induced by SA. This pathway regulates root growth and is nonrandomly distributed across *Massilia* species. Finally, we show that M117 colonization enriches specific bacterial taxa within roots. Our findings reveal a specific pathway employed by rhizosphere bacteria to colonize roots and promote their growth and highlight a useful microbial strategy and information for balancing host immunity and growth.

## Introduction

Roots are the primary organs of plants that take up nutrients and water from the soil [1], and they directly contact soil microbial communities and respond accordingly. Increasing evidence indicates that root-associated microbes can regulate root growth and development by secreting growth hormones or interfering with hormone biosynthesis and signaling in plant roots. Most beneficial microbes promote root growth by secreting auxin [2, 3]. However, root growth is precisely regulated by auxin in a concentration-dependent manner, and extremely high auxin levels inhibit root growth. Recent reports indicate that bacterial degradation of auxin plays a key role in growth promotion and is necessary for efficient rhizosphere colonization [4, 5]. In addition to auxin, many bacterial strains can modify the ethylene balance or ethylene response in roots, thereby affecting root system development [6–8], or suppress plant responses to abscisic acid to maintain plant mineral nutrient homeostasis [9]. These findings highlight the crucial roles of plant hormones in microbe-mediated root growth and development.

Salicylic acid (SA) is an essential plant hormone that plays critical roles in shaping plant immune responses to pathogens [10]. Under normal growth conditions, SA is present at low basal levels, but SA accumulates after pathogen infection [11], which can activate resistance gene expression in an NPR1-dependent manner. SA can lead to increased plant resistance but usually has a negative effect on plant growth [12, 13]. Therefore, SA-induced immune responses contribute to a growth–immunity trade-off [14]. In *Arabidopsis thaliana,* mutants such as *dnd1* and *agd2* [15, 16], which exhibit constitutively activated immunity and excessive SA accumulation, exhibit strongly stunted or dwarf phenotypes. Conversely, the *Arabidopsis sid2* and *S3H* mutants, whose SA levels are low, grow larger than the wild type plants [17, 18]. The overexpression of bacterial salicylate hydroxylase (*NahG*) can partially or fully reverse the dwarf phenotype of immune-activated mutants [19–21]. Indeed, the exogenous application of SA inhibits root and shoot growth [22, 23]. SA acts as a signaling molecule to modulate plant developmental signaling pathways [12, 24], but whether and how SA is involved in microbiota-mediated root growth remain unclear.

We recently discovered that the *Massilia* strains, M16 and M117, which were isolated from the soybean rhizosphere, promote root growth in a manner that depends on the level of external nitrogen [25]. A relationship between these *Massilia* strains and root systems has also been observed in maize, depending on the genotype and nitrogen levels [26, 27]. However, the mechanism by which *Massilia* regulates the growth of roots remains elusive. In this work, we revealed that *Massilia* significantly inhibits the root immune response of soybean. We discovered that *Massilia* possesses multiple SA degradation pathways, which are involved in the promotion of root growth. Moreover, we found that *Massilia* acquired genes related to SA degradation from other bacteria, which may be associated with their plant niche adaptations. Finally, we showed that *Massilia* can alter root-associated bacterial communities. Our findings extend understanding of how rhizosphere bacteria promote root growth and highlight the crucial role of bacterial SA hydrolysis capacity in plant–microbiota interactions.

## Materials and methods

### Root growth and bacteria quantification assays in water agar

Soybean seeds (DN50) were surface-sterilized with chlorine gas for 12 h, after which they were placed in sterilized glass dishes with filter paper. The *Massilia* strains M117 and M16, which were obtained from soybean rhizosphere soil, were used in this study [25]. These strains were cultured overnight at 28°C at 180 rpm using TY medium. The bacterial colonies were collected by centrifugation at 5000 r min^−1^ for 5 min and then resuspended in sterile ddH_2_O and diluted to a final OD_600_ of 0.08. Seven milliliters of bacterial suspension was added to each glass dish containing seeds for three days of germination at 25°C, and the same volume of sterile ddH_2_O was used as the control. Seedlings with consistent germination were selected and transplanted into tissue culture bottles containing water agar and grown under a 16 h photoperiod at 20°C at night and 28°C during the day. Different concentrations of SA or H_2_O_2_ were added to solidified water agar as needed, with DMSO or H_2_O serving as a solvent control. After 4–5 days of growth, the primary root length (from the root tip to the hypocotyl junction) of each seedling was measured with a ruler. To quantify bacterial colonization, deoxyribonucleic acid (DNA) was extracted from the root samples and quantitative polymerase chain reaction (qPCR) was performed by using the *Oxalobacteraceae*-specific primers Oxal_225f and Oxalo_656r [28]. This pair of primers has been demonstrated to efficiently amplify the DNA of the two *Massilia* strains used in this study (Fig. S1). The copy numbers of the samples were determined using strain-specific DNA standards.

### Transcriptome analysis

Experiments consisted of three treatments: water only (control) and inoculation with M117 or M16. Each treatment consisted of 4 replicates, with 2–3 seedlings in each replicate. We collected root samples from plants grown in water agar for 4–5 days following the experimental method described above. Ribonucleic acid (RNA) sequencing and data analysis were performed according to the methods described in our previous study [25]. Total RNA was extracted from the root samples using TRIzol reagent (plant RNA purification reagent for plant tissue) according to the manufacturer’s instructions (Invitrogen), and the genomic DNA was removed using DNase I (TaKaRa). RNA purification, reverse transcription, and library construction and sequencing were performed at Majorbio Biopharm Biotechnology Co., Ltd. (Shanghai, China) according to the manufacturer’s instructions (Illumina, San Diego, CA, USA). The sequenced reads were subsequently mapped to the *Glycine max* Wm82. a2. v1 reference genome using HISAT2 (http://ccb.jhu.edu/software/hisat2/index.shtml) software. Differential expression analyses were performed using the DESeq2 package. Genes that presented at least a 2-fold change in expression and an FDR ≤ 0.05 were considered differentially expressed genes (DEGs). RSEM (http://deweylab.biostat.wisc.edu/rsem/) was used to quantify the gene abundance. Gene Ontology (GO, http://www.geneontology.org) and Kyoto Encyclopedia of Genes and Genomes (KEGG, http://www.genome.jp/kegg/) enrichment analyses were carried out via GOtools and KOBAS.

### Reverse transcription–quantitative polymerase chain reaction analysis

Root samples (each treatment consisted of 3 replicates, with 2–3 seedlings per replicate) were collected as described above. Total RNA was extracted using TRIzol reagent, quantified using a NanoDrop 2000 (Thermo Fisher Scientific), and reverse-transcribed to complementary DNA (cDNA) using Hifair RII 1st Strand cDNA Synthesis SuperMix (YEASEN, CA, USA) according to the manufacturer’s instructions. Reverse transcription**–**quantitative PCR (RT-qPCR) was performed with SYBR Green PCR master mix (YEASEN, CA, USA) on a CFX96 real-time PCR detection system (Bio-Rad). The qPCR cycling conditions were as follows: initial denaturation for 30 s at 95°C, followed by 40 cycles of denaturation at 95°C for 10 s and annealing at 60°C for 45 s. The transcript level was calculated using the standard curve method from duplicate data, with the soybean *GmELF1b* (Glyma.02G276600) gene acting as the internal control [29]. The primers used for SA signaling-related genes in this study are listed in Table S1 [30].

### *Arabidopsis* growth assay

*A. thaliana* plants used in this study were in the Columbia-0 background. The *npr1–1* seeds were kindly provided by Shunping Yan [31]. The *sid2* and *s3hs5h* seeds were kindly provided by Kewei Zhang and Qiao Zhao [32]. Seeds were sterilized with sodium hypochlorite and stratified at 4°C in the dark for 2 days and then plated on MS medium. After growing for 2–3 days, when the roots reached approximately 1 cm in length, the seedlings were transferred onto Hoagland agar plates containing normal nitrogen nutrient, with eight seedlings placed onto each plate. Growth continued until the roots reached approximately 2 cm in length, at which point four seedlings on each plate were selected and inoculated next to their roots with 1 μL of a bacterial suspension at an OD_600_ of 0.0001. Each genotype included three replicates (plates). The plants were grown in growth chambers at 22°C under a 16 h light/8 h dark cycle. Root lengths were measured after 5–6 days of growth.

### Salicylic acid treatment and hormone quantification in vermiculite

Soybean seeds that were sterilized by chlorine gas were germinated on sterile water agar for 3 days. The seedlings were subsequently transplanted into pots (13 × 13 cm) with vermiculite, each of which contained 100 ml of either H_2_O, a suspension of *Massilia* M16, or a suspension of *Massilia* M117 (OD_600_ = 0.1), with one seedling per pot. After two days of growth, each of the three treatment groups (i.e., H_2_O, M16, and M117) were divided into two subgroups. One subgroup was irrigated with 50 ml of an aqueous solution containing 2 mM SA (a concentration that significantly affected soybean root growth under vermiculite conditions). The other subgroup was irrigated with 50 ml of an aqueous solution containing an equivalent volume of DMSO. There were a total of 6 treatments, with 15 seedlings per treatment. The plants were grown under a 16 h photoperiod at 20°C/28°C (night/day) and watered with sterilized H_2_O as needed. After 15 days of growth, the root lengths of the seedlings were measured, and photographs were taken. The roots were then excised. For each treatment, four biological replicates were prepared, each by pooling the roots from three seedlings (for a total of 12 seedlings per treatment). These samples were rapidly frozen in liquid nitrogen and subsequently sent to Wuhan Maiwei Biotechnology Co., Ltd. (Wuhan, China) for quantification of the SA and SA 2-O-β-glucoside (SAG) contents. Approximately 50 mg of each sample was rapidly frozen in liquid nitrogen, homogenized into a powder, dissolved in 70% methanol, vortexed, and placed at 4°C overnight. The contents of SA and SAG were subsequently analysed using a UPLC–MS/MS platform (UPLC, ExionLC AD; MS, Applied Biosystems 6500 Triple Quadrupole). Data acquisition was performed using Analyst 1.6.3 software (Sciex) to determine analyte responses on the basis of peak area integrations relative to the internal standard (Tables S2 and S3). The samples were then quantified using standard curves to determine their concentrations, which are expressed as ng/g fresh weight (FW).

### Bacterial growth phenotype, salicylic acid and pyruvate detection

Four *Massilia* strains (M16, M117, M12, and M22) were individually inoculated in 5 ml of minimal medium (MM) and incubated overnight at 28°C with shaking at 180 rpm. After centrifugation, each bacterial culture was separately resuspended in ddH_2_O to a final OD_600_ of 0.1. TY plates containing 0 mM (DMSO control), 0.5 mM, 1.0 mM, or 2.0 mM SA were prepared. After solidification, serial 10-fold dilutions (from 10^−1^ to 10^−4^) of each bacterial suspension were prepared in ddH_2_O, and 1 μL of each dilution was spotted onto the TY plates prepared as described above. Each treatment concentration included four replicates (plates). The plates were incubated at 28°C for 36–48 h and photographed to observe colony growth. For SA and pyruvate detection, *Massilia* strains M16 and M117 were separately inoculated into 5 ml of TY or MM liquid medium with or without SA, with four replicate tubes per strain. After incubation for various periods, the cultures were centrifuged to pellet the bacterial cells and collect the supernatant. The SA content in the supernatant was then determined as described above. The pyruvate content in the supernatant was quantified using a Pyruvate Assay Kit (Sangon Biotech Co., Ltd.).

### Genome sequencing analysis

*Massilia* strains M16 and M117 were inoculated into 5 ml of TY medium and incubated at 28°C for 24 h. DNA was then extracted using the GenElute Bacterial Genomic DNA Kit (Merck) according to the manufacturer’s instructions, and the concentration and quality of the DNA were confirmed via spectroscopy with a NanoDrop instrument. The genome was sequenced using a combination of Illumina sequencing and the Nanopore PromethION System. The raw Illumina sequencing reads that were generated from the paired-end library were subjected to quality filtering using fastp v0.23.0. Nanopore reads were extracted, subjected to basecalling and demultiplexed, and trimmed with a minimum Q score cut-off of 7. Afterwards, the clean short and long reads were assembled to construct complete genomes using Unicycler v0.4.8. As a final step, Unicycler uses Pilon v1.22 to polish the assembly using short-read alignments, reducing the rate of small errors. The coding sequences (CDSs) of the chromosomes and plasmids were predicted using Glimmer version 3.02 and GeneMarkS version 4.3 software. In accordance with the step-by-step instructions of BlastKOALA (https://www.kegg.jp/blastkoala/), the amino acid sequences (in fasta format) of the two bacterial strains were assigned to KEGG functions and their metabolic pathways were reconstructed. Briefly, using the reference GENES dataset for prokaryotes, functional annotation and classification were performed via BLAST search, followed by pathway reconstruction through KEGG Mapper [33].

### Gene expression analysis and nontargeted metabolomics study

*Massilia* strains M16 and M117 were inoculated into 5 ml of TY medium supplemented with either 0.1 mM or 1 mM SA (concentrations representing conditions with no effect or only slight growth inhibition, respectively). TY medium containing the same volume of DMSO served as the control. Therefore, the experiment consisted of a total of six treatments, each with four replicates. After 24 h of incubation at 28°C and 180 rpm, cells from both strains were collected. Total RNA was extracted using the RNAprep Pure Cell/Bacteria Kit (Tiangen, Beijing, China) and reverse-transcribed into cDNA using Hifair RII 1st Strand cDNA Synthesis SuperMix (YEASEN, CA, USA). RT-qPCR was performed with SYBR Green PCR master mix (YEASEN, CA, USA) on a CFX96 real-time PCR detection system (Bio-Rad). The transcript levels of the target genes were normalized to that of *gyrA*. The primers used in this study are listed in Table S4.

For the nontargeted metabolomic analysis, three SA concentrations (e.g. 0, 0.1, and 1 mM) were used. Additionally, to exclude any influence from the medium, we also tested TY medium without SA or *Massilia* inoculation. In total, seven treatments were established, and the TY-only treatment contained four replicates, whereas the other six treatments each contained six replicates. After 24 h of incubation at 28°C and 180 rpm and centrifugation, the supernatant was filter sterilized with a 0.22 μm membrane and then subjected to nontargeted metabolomics detection using a Thermo UHPLC-Q Exactive HF-X System equipped with an ACQUITY HSS T3 column (100 mm × 2.1 mm i.d., 1.8 μm; Waters, USA). The mass spectrometry data were collected using a Thermo UHPLC-Q Exactive HF-X mass spectrometer equipped with an electrospray ionization (ESI) source operating in positive and negative ion modes. Metabolite identification, annotation, and data analysis were performed by Majorbio Biopharm Technology Co., Ltd. Principal component analysis (PCA) with 95% confidence ellipses (based on unit-variance scaled data) combined with Adonis analysis was performed to assess the overall differences in metabolites between groups and the magnitude of variation within each group. The identified metabolites that were identified were annotated via the KEGG compound database, and the annotated metabolites were subsequently mapped to the KEGG pathway database (http://www.kegg.jp/kegg/). A heatmap analysis was conducted via MeV.v.4.8.1 software to visualize the relative levels and changes in metabolites. Data were normalized by row. Hierarchical clustering was then performed using Euclidean distances. The resulting heatmap was plotted with a red-blue color scheme and a fixed color scale ranging from −2 to 2.

### Tracking salicylic acid degradation using deuterium-labeled [^2^H6]-salicylic acid

Deuterium-labeled [^2^H6]-SA (SA-D6) was obtained from MedChemExpress LLC. SA-D6-based tracing experiments were conducted on the basis of a method from the literature [34] with slight modifications. The M117 and M16 strains were separately inoculated into TY medium and cultured for 12 h. The cells were harvested by centrifugation, washed once with fresh TY medium, and resuspended to an OD of 1.0. One milliliter of each suspension was then transferred to 5 ml of TY medium containing SA-D6 for isotope tracing. For each strain, four replicates were prepared. After 24 h of cultivation, 2 ml samples were collected, rapidly frozen in liquid nitrogen for 30 s, and quenched. The samples were then centrifuged at 4°C and 4000 × g for 3 min. The resulting cells were collected and the D6 metabolites were detected by LC–MS/MS(AB 6500+, AB Sciex), targeting parent and daughter ions specific to the D6 forms of the metabolites. The chromatographic conditions were as follows: flow rate, 0.2 ml/min; column, ACQUITY UPLC HSS T3, 100 Å, 1.8 μm, 2.1 mm × 150 mm; Precolumn, ACQUITY UPLC HSS T3 VanGuard, 100 Å, 1.8 μm, 2.1 mm × 5 mm; column temperature, 40°C; and mobile phase, solvent A: 0.1% formic acid in water; and solvent B: 100% acetonitrile. Gradient elution was performed as follows: 1 min, 5% B, 7 min, 95% B; 8.5 min, 95% B; 8.8 min, 5% B; and 12 min, 5% B. The abundances of the labeled and unlabeled metabolites are indicated as the peak areas of the spectra.

### Functional and phylogenetic analysis of the *NagGHAaAb* gene cluster

To heterologously express the *NagGHAaAb* gene cluster in rhizobia (*Sinorhizobium* CCBAU 45436), we followed a reported method [35] by inserting sequences encoding 2A peptides between the four SA-degrading genes, thereby fusing them into a single open reading frame expressed using a single promoter and terminator (Fig. S2). This expression cassette was synthesized by Nanjing GenScript Biotech Co., Ltd and then cloned into the pBBR1 vector. The verified pBBR vector carrying the correct construct was subsequently introduced into CCBAU 45436 via conjugation with the help of the helper plasmid pRK2013, following a previously described method [36]. The heterologous expression strains were confirmed by colony PCR and Sanger sequencing. The tolerance of the overexpression strains to SA and their effects on soybean root growth were assessed following the methods described above.

Gene cluster visualizations were generated using Clinker [37]. The analysis was performed on six *NagGHAaAb* gene clusters that were downloaded from the NCBI (accession numbers: AF036940, KX155564, DQ167474, GQ184726, AF039533, and AB237655). To estimate the evolutionary relationships of the *Massilia* strains, a phylogenetic tree based on the housekeeping gene sequences of 60 strains was constructed using the maximum likelihood method in IQ-TREE with 1000 bootstrap iterations, with *Burkholderia* sp. CCGE1003 designated as the outgroup. Fifty-eight *Massilia* genomes were downloaded from the NCBI (Table S5).

### Bacterial 16S ribosomal ribonucleic acid gene sequencing

Chlorine gas-sterilized seeds (DN 50) were germinated on sterile water agar for 3 days. The plants were subsequently transplanted to mixed soil (soil:vermiculite; 3:1 in volume) with 100 ml suspensions of *Massilia* M16 or M117 (OD_600_ = 0.1) in pots (13 cm × 13 cm) to ensure one seedling per pot. Soil treated with 100 ml sterile water was used as a control. After 7 days of growth, the rhizosphere soil and root samples were collected as described previously [38], after which the root lengths were determined as described above. Each treatment consisted of five replicates. Additionally, soils without plants were inoculated with or without *Massilia*, and bulk soil samples were collected after seven days. In total, 48 samples (15 rhizosphere soil, 15 root, and 18 bulk soil samples) were used for sequencing according to standard protocols at Majorbio Bio-Pharm Technology Co., Ltd. (Shanghai, China). Total microbial genomic DNA was extracted from 30 samples using an E.Z.N.A. soil DNA Kit (Omega Bio-Tek, Norcross, GA, USA). The DNA quality and concentration were determined by 1.0% agarose gel electrophoresis and a NanoDrop 2000 spectrophotometer (Thermo Scientific, USA), and the DNA was stored at −80°C until further use. The V5**–**V7 target regions of the 16S rRNA gene fragments were amplified with the primer pairs 799F (5′-AACMGGATTAGATACCCKG-3′) and 1193R (5′-ACGTCATCCCCACCTTCC-3′) [39] and sequenced on a NextSeq 2000 System (Illumina, San Diego, USA). After demultiplexing, the resulting sequences were filtered for quality with fastp (0.19.6) and merged with FLASH (v1.2.11). Afterwards, the high-quality sequences were denoised using the DADA2 plugin in the QIIME2 pipeline (version 2024) with the recommended parameters; maxEE was set to 2, which enables single-nucleotide resolution on the basis of the error profiles of the samples. DADA2-denoised sequences are usually called amplicon sequence variants (ASVs). The sequence data were rarefied to the lowest number of sequences per sample (43954). Taxonomic assignment of ASVs was performed using the Naive Bayes consensus taxonomy classifier at a 70% confidence level implemented in QIIME2 and the SILVA 16S rRNA database (v138). The Shannon index was calculated with Mothur v1.30.1. Principal coordinate analysis (PCoA) based on Bray–Curtis dissimilarity and a permutational multivariate analysis of variance (PERMANOVA) test (Adonis function, 999 permutations) were conducted to assess the percentage of variation associated with the treatment and statistical significance using the Vegan v2.4.3 package. For the Kruskal–Wallis H test, the false discovery rate (FDR) correction was used to identify significantly different taxa among the three treatments at the genus level.

### Statistical analysis

Differences among the three groups were analysed using the Kruskal–Wallis test. Comparisons of the control and *Massilia* strain treatments were performed by nonparametric Mann–Whitney tests, which were reported to be suitable for statistical testing of small sample size [40]. Comparisons of more than two groups were performed by one-way ANOVA followed by the Newman–Keuls test. Graphical representations were generated with GraphPad Prism 8 (GraphPad Software, Inc., La Jolla, CA, USA). The means and standard deviations (SDs) of the data were calculated.

## Results

### *Massilia* inhibits SA-related immune signaling in roots

**Figure 1:**
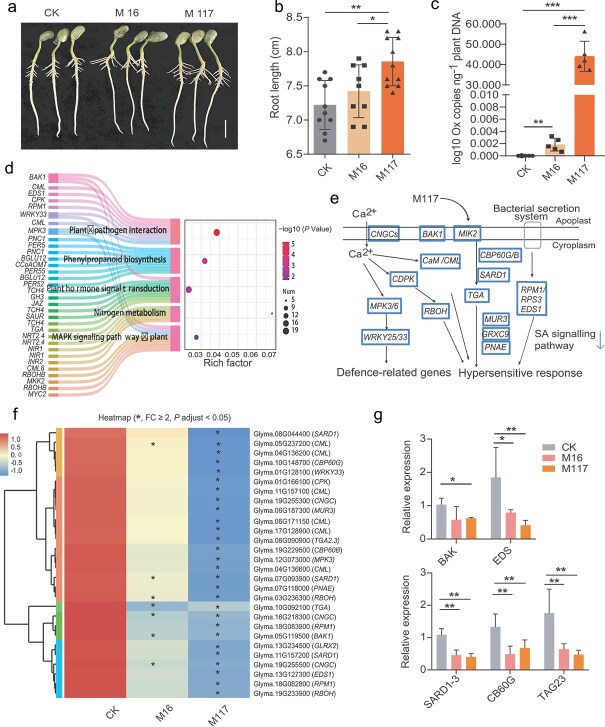
*Massilia* inhibits SA-related immunity signaling in roots. (a) Effects of *Massilia* on the growth of soybean roots. Bar = 2 cm. (b) Lengths of the soybean roots shown in (a). The bars depict the averages of 9–10 replicates. (c) The abundances of *Massilia* in soybean roots are shown in (a). The bars depict the averages of five replicates. (d) Representative KEGG pathway analysis of genes that were downregulated in response to M117 treatment. *P* < 0.05. (e) Genes related to the defense and SA signaling pathways that are significantly downregulated in M117. (f) Expression heatmaps of defense and SA signaling pathway-related genes in the roots of the plants treated with or without *Massilia*. The gene annotations and numerical values can be found in Dataset S1. (g) The relative expression levels of immune genes in response to different treatments were quantified via RT–qPCR. Means ± SDs, *n* = 4 and with three technical replicates. Statistical analyses were performed via one-way ANOVA followed by the Newman–Keuls test, and significance is denoted by asterisks, where ^*^ indicates *P* < 0.05 and ^***^ indicates *P* < 0.001.

To investigate the mechanism by which *Massilia* promotes root elongation, we performed a water agar growth test with two strains of *Massilia*, namely, M16 and M117. Compared with the control and M16 treatments, the M117 treatment significantly promoted the growth of primary roots (Fig. 1a and b) and the colonization abundance of M117 in the roots was significantly greater than that of M16 (Fig. 1c). PCA revealed that the gene expression profiles of M117- and M16-treated roots clustered together and were separated from those of control cluster (Fig. S3a). However, differential gene analysis (*P* adjusted <0.05; FC ≥ 2) revealed that M117 treatment resulted in 704 DEGs in plant roots, which was more than twice the number (391 DEGs) induced by the M16 treatment. Among these, 213 DEGs were common to both the M117 and M16 treatments, whereas 527 and 178 unique DEGs were specific to the M117 and M16 treatments, respectively (Fig. S3b, Datasets S1 and S2).

KEGG pathway enrichment analysis of the DEGs was subsequently performed. Similarly, the “glycolysis/gluconeogenesis” pathway was significantly enriched among the upregulated DEGs in M117-treated roots [25]. However, we found that the “plant–pathogen interaction” and “plant hormone signal transduction” pathways were significantly suppressed by both *Massilia* strains (Fig. S3C). Many defense-related genes, such as *CNGC*, *CML*, *CPK*, *BAK1*, *MIK2*, *RBOH*, *MPK3*, *WRK33*, *EDS1*, and *RPM1,* were strongly downregulated in response to M117 treatment. Furthermore, many genes associated with the SA signaling pathway (including *PNAE*, *GRXC*, *GLRX*, *GLRX2*, *SARD1*, *CB60G*, *CB60B*, *MUR3*, *TGA10*, and *TAG23*) were also significantly downregulated (Fig. 1d and e). Among these 28 genes, *SARD1* (0.133) and *CB60G* (0.137), which encode master transcription factors involved in SA production and the immune response [41, 42], presented the greatest differences in expression between *Massilia*-treated and untreated roots (Dataset S1). Among the 28 immune-related genes mentioned above, only eight, including *CB60G* and *TAG*, were significantly suppressed in the samples treated with M16 (Fig. 1f). RT-qPCR analysis confirmed significant changes in the expression of several genes and revealed that both strains significantly suppressed the expression of *EDS1*, *SARD1–3*, *CB60G*, and *TAG23* in plant roots, with the exception of *BAK1* (Fig. 1g). These results suggest that both *Massilia* strains can inhibit SA-related immune signaling, but M117 has a stronger suppressive effect on immune-related genes than M16 does.

### *Massilia*-mediated root elongation depends on the salicylic acid signaling pathway

**Figure 2:**
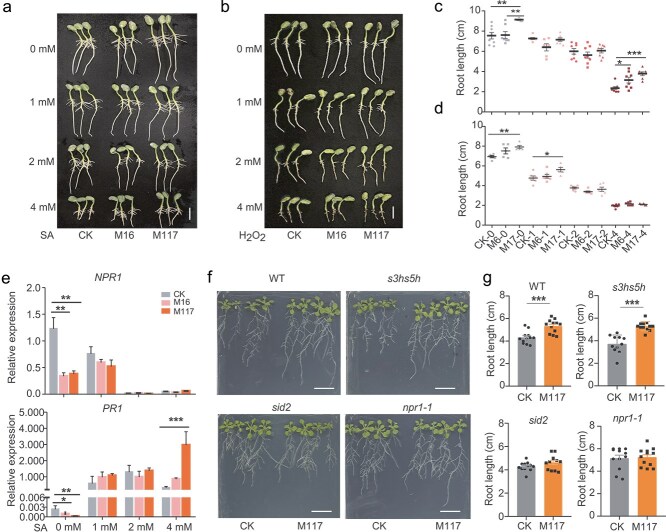
*Massilia*-mediated root elongation is associated with SA and the SA signaling pathway. (a) Effects of SA on *Massilia*-mediated soybean root elongation. Bar = 2 cm. (b) Effects of H_2_O_2_ on *Massilia*-mediated soybean root elongation. Bar = 2 cm. (c) Root lengths of the soybean roots shown in (a). (d) Root lengths of the soybean roots shown in (b). The bars depict the averages of seven to eight replicates. Statistical analyses were performed via one-way ANOVA followed by the Newman–Keuls test, and significance is denoted by asterisks, where ^*^ indicates *P* < 0.05, ^**^ indicates *P* < 0.01, and ^***^ indicates *P* < 0.001. (e) The relative expression levels of *NPR1* and *PR1* under different treatments were determined by RT–qPCR. Means ± SDs, *n* = 4 and with 3 technical replicates. Statistical analyses were performed via one-way ANOVA followed by the Newman–Keuls test, and significance is denoted by asterisks, where ^*^ indicates *P* < 0.05 and ^***^ indicates *P* < 0.001. (f) The effect of M117 on root growth of *Arabidopsis* WT, *s3hs5h, sid2*, and *npr1–1* mutants. Bar = 2 cm. (g) Root lengths of the *Arabidopsis* roots shown in (f). The values are the means ± SDs (*n* = 12). Statistical analyses were performed via Mann–Whitney nonparametric tests, and significance is denoted by asterisks, where ^*^ indicates *P* < 0.05.

Given the critical role of SA in mediating the growth-defense trade-off [14], we proceeded to evaluate the effect of exogenously applied SA on root growth mediated by the two strains. Compared to the control group without SA, *Massilia* M117-induced root elongation was significantly attenuated upon supplementation with 1 mM or 2 mM SA (Fig. 2a and c). However, roots treated with the *Massilia* strains M16 and M117 were more tolerant to high concentrations of SA (4 mM). These results indicate that *Massilia* can mitigate the inhibitory effect of high SA concentrations on root growth. Additionally, we tested the ability of *Massilia* to alleviate stress from reactive oxygen species by adding H_2_O_2_ to the water-agar medium. M117 still promote soybean root growth at low H_2_O_2_ concentrations, but both strains failed to alleviate the root growth inhibition caused by high H_2_O_2_ concentrations (Fig. 2b and d). Thus, *Massilia* exhibited different responses to SA and H_2_O_2_, suggesting that *Massilia*-mediated root growth is associated with SA. Correspondingly, the SA response marker genes *NPR1*, *PR1*, and *PR2* were significantly downregulated following treatment with M16 and M117. Although the three genes showed distinct response patterns to SA supplementation, exogenous SA treatment effectively alleviated the inhibitory effects of both strains on the expressions of these genes within a certain concentration range (Fig. 2e and Fig. S4).

To further explore the relationship between *Massilia*-mediated root growth promotion and SA signaling, we examined the responses of *Arabidopsis* mutants that overaccumulated SA (*s3hs5h*), were defective in SA biosynthesis (*sid2*), and were impaired in SA perception (*npr1*) to strain M117. M117 promoted root elongation in the *s3hs5h* mutant, whereas its ability to promote root elongation in the *sid2* and *npr1* mutants was significantly attenuated (Fig. 2f and g). These findings suggest that M117-mediated root elongation is dependent on the SA signaling pathway. Taken together, these results suggest that M117 promotes root elongation through modulating the SA signaling pathway.

### *Massilia* reduces the salicylic acid content in roots and exhibits strain-specific for salicylic acid tolerance and degradation capacities

**Figure 3:**
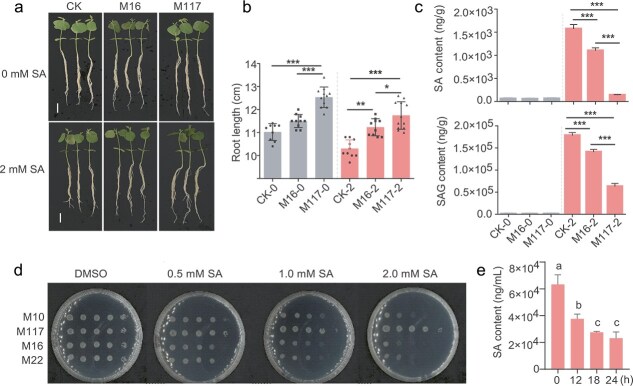
*Massilia* reduces the contents of SA and SAG in roots and displays strain-specific SA tolerance. (a) Effects of SA on *Massilia*-mediated soybean root elongation after vermiculite treatment. Bar = 2 cm. (b) Root lengths (*n* = 10–12) of the seedlings shown in (a). (c) Contents of SA and SAG (*n* = 4) in the seedlings shown in (a). Statistical analyses were performed via one-way ANOVA followed by the Newman–Keuls test, and significance is denoted by asterisks, where ^*^ indicates *P* <0.05 and ^***^ indicates *P* < 0.001. (d) Effects of different concentrations of SA on the growth of *Massilia* on MM plates. A representative plate from three plates is shown. (e) Time-course analysis of SA degradation by M117. The values are the means ± SDs (*n* = 3). Statistical analyses were performed via one-way ANOVA followed by the Newman–Keuls test, and different lowercase letters indicate significant differences (*P* < 0.05).

Because *Massilia* can alleviate the inhibitory effect of high SA concentrations on root growth, we hypothesized that *Massilia* may reduce SA levels in roots. We thus measured SA concentrations in the roots treated with *Massilia* and control plants in the absence or presence of 2 mM SA under vermiculite cultivation conditions. Consistent with the above results, only M117 promoted root growth after 15 days of normal growth in vermiculite. However, in the presence of SA, both M117 and M16 promoted root growth after 15 days, but the increase in root length following M117 treatment was more pronounced (Fig. 3a and b). Under normal conditions, SA levels in roots are low, and no significant changes were detected in root SA contents between the treatments with the two bacterial strains and the control. Nevertheless, in the presence of 2 mM SA, the SA and SAG levels increased significantly (26.3- and 216-fold, respectively) in the control roots. However, the increases in SA and SAG levels in roots inoculated with M16 and M117 were much lower than those measured in control roots, with 20.2- and 180.2-fold and 2.24- and 64.4-fold increases after M16 and M117 treatment, respectively (Fig. 3c). Thus, the two *Massilia* strains reduced SA levels in roots, with the M117 strain exhibiting a stronger reduction capacity.

We subsequently grew these two strains, alongside two other strains (M10 and M22), on MM containing different concentrations of SA. In the absence of SA, all the strains grew well and similarly. However, as the SA concentration increased from 0.5 to 2 mM, the four strains exhibited different sensitivities to SA. At 2 mM SA, M117 exhibited optimal growth with minimal growth inhibition, whereas the growth of the remaining three strains was strongly inhibited (Fig. 3d). This suggests that M117 may be able to degrade SA. To test this hypothesis, we first added 0.5 mM SA to liquid MM medium and monitored the growth of M117 and the residual SA content in the supernatant after 12, 18, and 24 h of cultivation. After 12 h of cultivation in medium containing SA, the OD value of strain M117 reached approximately 0.20, showing no significant difference from the control without SA, with an SA degradation rate of 40.7%. As the cultivation time extended, the degradation rate further increased to 56.5% after 18 h. Following 24 h of cultivation, the OD value of the SA-treated group reached 0.39, slightly lower than that of the control (0.44); nonetheless, the strain still achieved an SA degradation rate of 63.5% (Fig. 3e, Fig. S5a). In addition, we further examined the degradation capacity of M117 at SA concentrations of 0.1 mM and 1 mM. After 24 h of cultivation, the degradation rates of M117 at these two concentrations were 91.4% and 31.2%, respectively (Fig. S5b). These results suggest that strain M117 is capable of degrading SA, with the highest degradation efficiency observed at a concentration of 0.1 mM SA.

### *Massilia* strains harbor genetically and functionally diverse pathways for salicylic acid degradation

**Figure 4:**
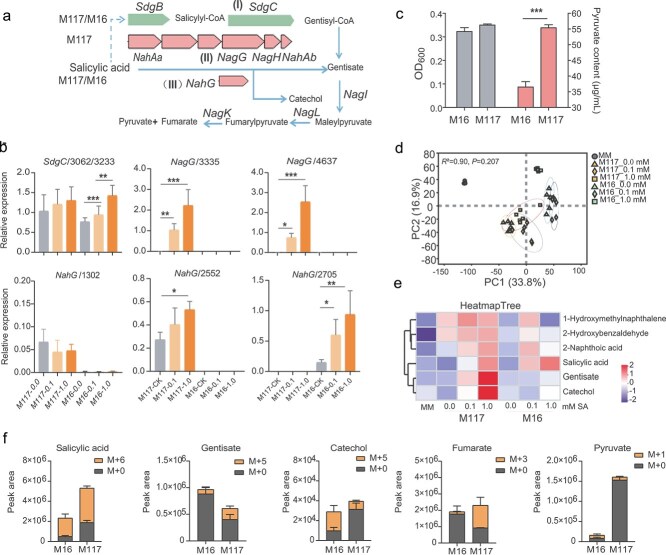
*Massilia* has diverse SA degradation-related genes. (a) Genes involved in the SA degradation pathway in the M117 and M16 genomes. (I) CoA-mediated gentisate pathway; (II) *Nag* gene cluster-mediated gentisate pathway; and III) catechol pathway. (b) The relative expression levels of SA degradation-related genes under different SA treatments were quantified by RT–qPCR. Means ± SDs, *n* = 4 and with three technical replicates. Statistical analyses were performed via one-way ANOVA followed by the Newman–Keuls test, and significance is denoted by asterisks, where ^*^ indicates *P* < 0.05 and ^***^ indicates *P* < 0.001. (c) M117 and M16 growth and pyruvate production in MM medium. The values are the means ± SDs (*n* = 4). Statistical analyses were performed via Mann–Whitney nonparametric tests, and significance is denoted by asterisks, where ^***^ indicates *P* < 0.001. (d) PCA of the metabolite profiles of M117 and M16 in MM after the addition of different concentrations of SA (*n* = 40). (e) Heatmap of naphthalene degradation pathway metabolites in MM after inoculation with M117 or M16 and the addition of different concentrations of SA. The colors (from dark blue to bright red) and the intensity of the shading indicate metabolite abundances, from low to high. (f) The spectral intensities of isotopically labeled SA and downstream metabolites in the bacterial cells of *Massilia* M117 and M16. The values are the means ± SDs (*n* = 4).

To elucidate the underlying mechanism of SA degradation by *Massilia*, we sequenced the whole genomes of the M117 and M16 strains. The general genomic features of these two strains are shown in Table S6. BlastKOALA annotation and alignment analysis revealed that M117 and M16 have different numbers of SA degradation pathways. Among them, the M117 genome contains genes that are associated with three SA degradation pathways: the first pathway (I) is via *sdgC* (salicyloyl-CoA 5-hydroxylase), which catalyzes the conversion of salicylyl-CoA to gentisyl-CoA [43]; the second pathway (II) involves the *NagGHAaAb* gene cluster (containing the salicylate 5-hydroxylase gene), which catalyzes the formation of gentisate from salicylate [44]; and the third pathway (III) is mediated by the *NahG* (salicylate 1-hydroxylase) gene for the conversion of SA to catechol (Fig. 4a) [45]. Furthermore, there were two copies of the *NagGHAaAb* gene cluster (inverted repeats) for pathway II and the *NahG* gene for pathway III in the M117 genome, and both copies of these genes were genetically distinct (Fig. S6a). In contrast, the M16 genome contains genes associated with only two SA degradation pathways (I and III), and only one *NahG* gene was found in pathway III (Table S7).

To investigate whether these genes are involved in SA degradation, we examined their responsiveness to SA. When we added varying concentrations of SA, the *SdgC* (3233) gene and the *NahG* (2705) gene were significantly induced in M16. However, in M117, the *NahG* gene (e.g. 2552) and the two *NagG* genes (e.g. 3335 and 4737) were all significantly induced, with the two *NagG* showing the strongest induction (Fig. 4b). These results indicate that both *Massilia* strains are responsive to SA and induce the expression of genes associated with SA degradation, with strain M117 exhibiting a stronger ability to induce the expression of its two *NagG* genes.

Gentisate and catechol are key intermediates in the aforementioned three pathways. However, gentisate can undergo further catalysis to form pyruvate through pathways I and II (Fig. 4a). Accordingly, we detected high levels of pyruvate in the supernatant cultures of M117 and M16, and the pyruvate level in M117 was significantly greater than that in M16 (Fig. 4c). However, the addition of low concentrations (0.01 and 0.1 mM) of SA further increased the pyruvate content in the supernatant of M16 but not in that of M117 (Fig. S6b). When the SA concentration reached 1 mM, the growth of M16 was significantly inhibited, whereas that of M117 was unaffected (Fig. S6c), again demonstrating that M117 is more tolerant to SA. Pyruvate is nonspecific central metabolite in many biochemical pathways [46]. To investigate other downstream metabolic products, untargeted metabolomics was performed. With increasing SA concentration, the metabolite profile of M16 significantly differed from that of M117 (Fig. 4d). The SA content in the M117 supernatant was lower than that in the M16 supernatant, with large amounts of gentisate and catechol detected in the former and very low levels of these compounds detected in the latter (Fig. 4e; Dataset S3).

To confirm the existence of these SA degradation pathways in M117, SA-D6 tracking experiments were conducted. After culturing *Massilia* in a medium containing SA-D6 for 24 h, we found that deuterium labeling from SA-D6 was incorporated into gentisate, catechol, fumarate, and pyruvate molecules and was detected in the cell extracts. The form of catechol that was labeled with five deuterium atoms (M + 5) showed the highest labeling abundance (66%) in strain M16, whereas the form of fumarate that was labeled (M + 5) showed the highest labeling abundance (58%) in strain M117. Although the pyruvate labeling ratio (M + 1) in strain M16 was relatively high at 48%, its total concentration was much lower than that in M117. Due to the large endogenous pool of pyruvate in strain M117, only about 5% was ultimately derived from the labeled SA (Fig. 4f). Together, these data further suggest that *Massilia* can degrade SA via the aforementioned pathways.

### *NagGHAaAb* gene cluster is involved in regulating root growth and is nonrandom distributed among *Massilia* species

**Figure 5:**
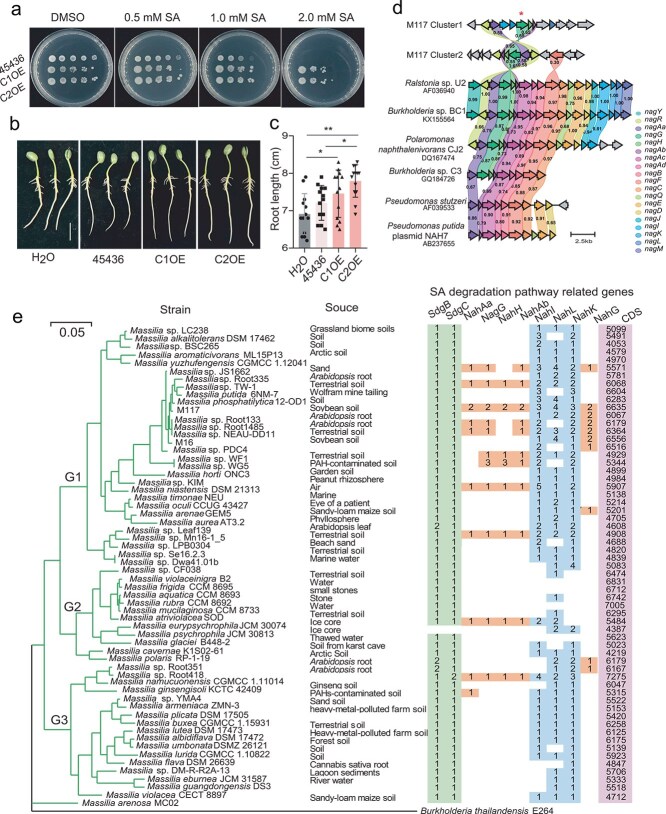
Phylogenetic tree, metabolic function, and distribution of SA-hydrolyzing genes in *Massilia*. (a) Growth of wild-type and *NagGHAaAb* gene cluster-overexpressing *Sinorhizobium* CCBAU 45436 (C1OE and C2OE) on MM plates containing different SA concentrations. A representative plate from 3 plates is shown. (b) Effect of wild-type and *NagGHAaAb* gene cluster-overexpressing CCBAU 45436 (C1OE and C2OE) on soybean root growth. Bar = 2 cm. (c) Root lengths of the soybean roots shown in (b). The bars depict the averages of 10–12 replicates. Statistical analyses were performed via one-way ANOVA followed by the Newman–Keuls test, and significance is denoted by asterisks, where ^*^ indicates *P* < 0.05, ^**^ indicates *P* < 0.01, and ^***^ indicates *P* < 0.001. (d) Conservation of the *NagGHAaAb* cluster identified in other fungi. ^*^ indicates the *NagG* gene. The numbers between proteins indicate amino acid identity. (e) Phylogenetic tree based on 31 housekeeping genes of 60 *Massilia* strains. The tree was constructed using the maximum likelihood method. The numbers at the nodes indicate the bootstrap values (in percentages) from 1000 replicates. The scale indicates similarity. The numbers within colored blocks indicate either gene counts or coding sequence (CDS) sizes.

Given that the M117 strain has an additional pathway that contains two copies of *NagGHAaAb* gene clusters, which are strongly induced by SA, we attempted to determine whether the stronger ability of this strain to promote root growth is due to SA degradation by the *NagGHAaAb* gene cluster. Due to the immature genetic manipulation system in *Massilia*, establishing a stable gene knockout system remains challenging. Therefore, we chose to heterologously overexpress these two gene clusters in soybean rhizobia to preliminarily analyse their promotion of root growth. Compared with the wild-type strain, the *NagGHAaAb-*overexpressing strain not only increased SA tolerance (Fig. 5a) but also significantly promoted soybean root growth (Fig. 5b and c). These results suggest that the *NagGHAaAb* gene cluster may confer SA-degrading activity, thereby promoting root growth.

The *NagGHAaAb* gene cluster has been shown to participate in naphthalene degradation and has been reported predominantly in *Ralstonia*, *Polaromonas*, and *Burkholderia* [44, 47, 48]*.* We then performed a homology comparison analysis on the *NagGHAaAb* gene clusters between M117 and these bacteria. We found that the *NagGHAaAb* gene cluster was well preserved and relatively conserved in *Ralstonia*, *Burkholderia,* and *Polaromonas*. However, the *NagGH* gene was lost in *Pseudomonas*. The genes in cluster 2 of M117 were similar to those in *Ralstonia* but exhibited low homology, ranging from 0.55 to 0.74 (Fig. 5d). The positions of the genes in cluster 1 changed, and there was low homology between the two clusters, suggesting that these two gene clusters may have distinct evolutionary origins and have undergone recombination or insertion events. A phylogenetic analysis of 125 homologous sequences (>78% identity to *NagG* in the UniProt database) from 12 families and 45 genera revealed that *NagG* sequences from different genera tended to cluster together, indicating the possibility of horizontal gene transfer. The two M117 *NagG* copies fell into distinct subbranches within *Oxalobacteraceae*. Furthermore, sequences from *Oxalobacteraceae* and *Alcaligenaceae* formed a larger clade, suggesting a relatively close ancestral lineage for *NagG* in these families (Fig. S7).

To further investigate the distribution of this gene cluster within *Massilia* species, we compared the genomes of strains M16 and M117 with those of 58 *Massilia* strains obtained from the NCBI database (Table S5). A phylogenomic tree based on their housekeeping genes revealed that the 60 strains could be categorized into three main groups (Fig. 5e). M117 and M16 were grouped together and located in the same subbranch of group I, along with multiple *Massilia* strains isolated from the roots of *Arabidopsis* [49]. We analysed the genes related to SA pathways in the genomes of these strains and found that 98% of the strains (59 strains) harbored *SdgC* genes (pathway I), indicating that this pathway is conserved in *Massilia.* However, pathways II and III were present in only some strains, most of which were in group I. Most of the strains in this group were isolated from plant roots or rhizosphere soil (Fig. 5e). These results suggest that the enrichment of SA metabolism genes in *Massilia* strains from plant-associated niches may facilitate their adaptation to the rhizosphere or root environment*.*

### *Massilia* efficiently colonizes the roots and alters the composition of the root bacterial community

**Figure 6:**
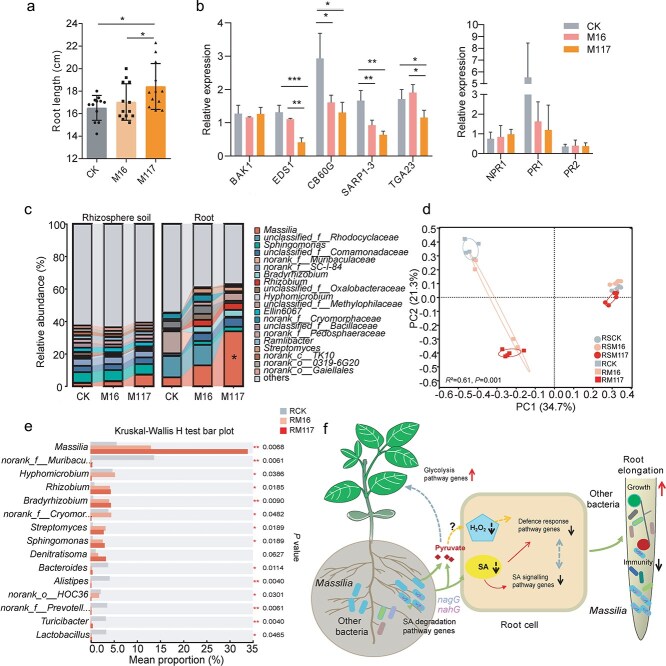
*Massilia* alters the composition of the root bacterial community. (a) Effects of *Massilia* on the growth of soybean roots under soil conditions. (b) The relative expression levels of immune genes in response to different treatments were quantified via RT–qPCR. Mean ± SDs, *n* = 4 and with three technical replicates. Statistical analyses were performed via one-way ANOVA followed by the Newman–Keuls test, and significance is denoted by asterisks, where ^*^ indicates *P* < 0.05 and ^**^ indicates *P* < 0.01. (c) Relative abundances at genus level of the top 20 taxa in root and rhizosphere samples across treatments; ^*^ indicates *Massilia.* (d) PCoA analysis based on Bray–Curtis distances of the root and rhizosphere samples across treatments. *n* = 30. (e) The different relative abundances of the top 15 genera in root samples among the three treatments. Significant differences between treatments were determined by the Kruskal–Wallis H test (*n* = 5). (f) Proposed model for *Massilia-*mediated root growth and bacterial colonization.

We next investigated the ability of *Massilia* to colonize the soybean rhizosphere and its impact on the composition of the associated bacterial community under soil conditions. In agreement with the results of the water agar assays, M117 significantly promoted root growth (Fig. 6a). In addition, the expression levels of the immunity-related genes *EDS1*, *CB60G*, *SARD1–3,* and *TAG23* decreased following treatment with M117. However, there was no significant change in the expression levels of *BAK1*, *NPR1*, *PR1,* and *PR2* (Fig. 6b)*.* The relative abundances of *Massilia* in the rhizosphere soil and in the roots increased after inoculation with M16 or M117, with the M117 treatment having a greater effect than the M16 treatment. In particular, the abundance of *Massilia* increased by 6.20- and 2.63-fold in the roots, respectively, compared with those in the untreated control and M16 treatments (Fig. 6c). Given that the expression of *BAK1*, *NPR1*, *PR1*, and *PR2* was downregulated after M117 treatment under sterile vermiculite conditions (Fig. S8), we speculate that under soil conditions, other microorganisms may interfere with the expression of these genes. Consistent with this hypothesis, inoculation with *Massilia* altered the bacterial community composition in the roots and rhizosphere (Fig. 6c), although it had little effect on species diversity (Fig. S9a). PCoA and PERMANOVA revealed significant changes in bacterial community composition in both the rhizosphere and roots (Fig. 6d, Table S8), with more pronounced differences observed in the roots. Among the top 15 genera, the relative abundances of 14 genera changed. For instance, the relative abundances of *Rhizobium*, *Bradyrhizobium*, *Streptomyces*, *Sphingomonas*, and *Phenylobacterium* increased, whereas those of *Bacteroides, Turicibacter,* and *Lactobacillus* decreased. In the rhizosphere soil, only three genera showed significant changes in relative abundance: the abundance of *Massilia* and *norank_o__Subgroup_7* increased, whereas that of *norank_c__TK10* decreased (Fig. 6e, Fig. S9b). These results indicate that *Massilia* is adept at colonizing roots and influencing the assembly of other root-associated bacteria.

To further investigate the direct effect of *Massilia* on soil bacterial communities, a supplementary inoculation experiment was conducted in bulk soil in the absence of plants. Under these conditions, inoculation with *Massilia* significantly reduced the species diversity (Fig. S9c). An increased abundance of *Massilia* was found to increase the abundances of other bacteria, such as *norank_o__Subgroup_7* and *Nitrospira* (Fig. S9d). These results suggest that *Massilia* can influence the abundances of other bacteria; however, the direction, intensity, and target genera of its effects depend on the ecological niche it inhabits.

## Discussion

Rhizobacteria have been widely reported to affect the root growth of their host plants. Previous studies have shown that one of the main reasons for this is the regulation of plant hormone levels, primarily involving auxin, ethylene, and abscisic acid [3, 8, 9]. *Massilia* is a widely distributed rhizosphere taxon that has recently been shown to contribute to the root development and growth performance of maize and soybean [25–27]. Here, we revealed a previously less highlighted mechanism by which *Massilia* promotes plant root growth by attenuating SA-dependent immune signaling.

Our water agar experiments confirmed that *Massilia* colonization significantly promoted root elongation within 5–6 days (Fig. 1a). Transcriptomic profiling revealed a notable downregulation of immune-related genes, particularly those involved in plant-pathogen interactions after M117 treatment (Fig. 1d). Among these, some key SA signaling genes, especially *SARD1* and *CB60G*, were suppressed after M117 treatment (Fig. 1g). Given that reduced SA levels and immune activities often positively correlate with increased plant growth [17, 18], these findings suggest that M117 likely stimulates root elongation through modulation of SA-mediated immunity. This hypothesis was further tested by exogenous SA application. At low concentrations, SA partially suppressed the root-promoting effect of M117, whereas at high concentrations, both M117 and even M16 retained the ability to promote root growth (Fig. 2a and c). Such dose-dependent responses align with known dual roles of SA in plant defense and development [23]. In *A. thaliana*, SA signaling at moderate to high concentrations (e.g. 150 μM to 1 mM) is largely regulated by NPR1, a central regulator of systemic acquired resistance [50]. We consistently observed that *Massilia* M117 significantly modulated the expressions of SA-responsive genes (e.g. *NPR1*, *PR1*, and *PR2*) under water agar culture conditions, reinforcing its role in inhibiting SA-related immune responses (Fig. 2e). Genetic evidence from *Arabidopsis sid2* and *npr1* mutants confirmed that a functional SA biosynthesis and signaling pathway are required for M117-mediated root elongation (Fig. 2f and g).

Both M117 and M16 were able to alleviate the inhibition of root growth caused by high concentrations of exogenous SA (Fig. 2a and c). Given that high concentrations of SA directly inhibit plant growth, we hypothesize that M117 and M16 may reduce the accumulation of SA in roots. Indeed, we observed a significant decrease in SA and SAG levels in plants that were inoculated with M117 and M16 compared with those in the control group when 2 mM SA was added under vermiculite culture conditions (Fig. 3c), and compared with M16, M117 could tolerate 2 mM of SA (Fig. 3d). Direct degradation assays showed that M117 efficiently metabolizes SA (Fig. 3e). Although microbial degradation of SA is well-documented in multiple genera, such as *Pseudomonas*, *Streptomyces*, *Rhizobium*, and the plant pathogenic bacteria *Ralstonia* [51–53], our genomic analysis revealed that M117 possesses a more extensive genetic repertoire for this function than M16. BlastKOALA annotation indicated that M117 has three distinct SA degradation pathways, and the genes in pathways II (*NagGHAaAb*) and III (*NahG*) have two copies. In contrast, M16 contains only two pathways, each represented by a single gene copy (Fig. 4a, Table S7). Transcriptional analysis showed that the two *NagG* homologs in M117 were strongly induced by SA (Fig. 4b), suggesting that they are primary contributors to its degradation capacity. Metabolomic profiling supported the activities of these pathways, showing SA-dependent enrichment of the downstream metabolites gentisate and fumarate, while isotopic tracing confirmed the metabolic fluxes from SA into these compounds (Fig. 4f). Although M117 can produce high levels of pyruvate (Fig. 4c), its pool was not substantially enriched by exogenous SA (Fig. S3b), and only a minor fraction of the pyruvate pool was derived from labeled SA (Fig. 4f). It is likely that the pyruvate levels may have reached saturation or that pyruvate was metabolized into downstream substances. Pyruvate can act as a scavenger of hydrogen peroxide and mitigate oxidative stress in other biological processes [54]. Whether pyruvate plays a similar role in M117-mediated immune suppression warrants further exploration.

For M117, the *NagGHAaAb* gene cluster was highly induced by SA, yet its direct role in SA tolerance and root growth promotion remains unclear. We heterologously expressed this cluster in a rhizobium, which conferred enhanced SA tolerance and promoted soybean root growth (Fig. 5a–c), indicating the functional capacity of the gene cluster in regulating root growth. Nevertheless, whether this cluster is the primary determinant of M117-mediated root growth promotion in its native context requires further investigation. A comparative analysis of *NagGHAaAb* gene clusters revealed that the gene clusters in *Massilia* M117 exhibited low homology with those previously reported in other bacteria (Fig. 5d)*.* Additionally, genomic analysis of *Massilia* species revealed that pathway I was conserved in *Massilia,* whereas pathways II and III were only present in some strains (Fig. 5e), which exhibited a non-random distribution across *Massilia* species. SA is an important hormone for plant growth and resistance to pathogens. There is evidence that *Ralstonia solanacearum* degrades plant SA to protect itself from inhibitory levels of this compound and also to increase its virulence on plant hosts [51]. Most M*assilia* strains that carry these accessory SA-degradation genes were isolated from the plant roots or rhizosphere soil (Fig. 5e). Collectively, these results suggest that these SA-degradation genes may facilitate the adaptation of *Massilia* to the rhizosphere niche, potentially by modulating host immune signaling to foster a commensal or beneficial interaction.

As a key immune signaling molecule, SA and SA-mediated immunity have been shown to regulate the colonization of specific bacterial taxa, such as *Pseudomonas* and *Streptomyces*, in the root and rhizosphere [55, 56] and to shape root-associated microbial communities in *Populus trichocarpa*, *Arabidopsis*, and rice [57–59]. Consistent with these findings, *Massilia* inoculation enriched several root-associated bacterial groups, including *Rhizobium*, *Bradyrhizobium*, *Streptomyces*, and *Sphingomonas* (Fig. 6e). This enrichment may be linked to the suppression of host immune signaling by *Massilia*. Under soil conditions, the expressions of immune-related genes, such as *EDS1*, *CBP60g*, *SARD1–3,* and *TAG,* remained suppressed. In contrast, the suppression of *BAK1*, *NPR1*, *PR1*, and *PR2* was alleviated in the natural soil environment (Fig. 6b). This shift likely results from the increased abundances of other root-colonizing bacteria, which may trigger plant immune responses and thereby partially counteract the immune-suppressive effect mediated by *Massilia*. This suggests that the relationship between *Massilia* colonization and SA signaling is dependent on the plant’s growth environment. Future studies should focus on the colonization dynamics of *Massilia* in roots and the rhizosphere, examine its interactions with other enriched bacteria, and evaluate whether these microbial shifts further influence plant growth and development.

In summary, our study reveals a new rhizobacterial mechanism that promotes soybean root growth by mediating the growth–immunity trade-off. In brief, *Massilia* strains harboring genetically diverse SA-degrading genes can efficiently colonize roots and the rhizosphere, reducing SA levels and/or producing pyruvate in the roots, which in turn attenuates root immune responses. This immunosuppression facilitates root growth and may further support the enrichment of *Massilia* and other specific bacteria in the root (Fig. 6f). Such an interaction reflects a co-evolutionary adaptation between plants and rhizosphere microbes, providing a mechanistic basis for future efforts to harness rhizobacteria for sustainable plant growth promotion.

## Supplementary Material

Supplementary_material_wrag082

## Data Availability

Raw data were deposited in the Genome Sequence Archive at the China National Genomics Data Center under the accession numbers CRA026523, CRA026402, CRA037932 and OMIX010485. *Massilia* M117 and M16 have been deposited in NCBI Sequence Read Archive (SRA) database with the accession nos. PRJNA1242737 and PRJNA1242688, respectively.
