# Supplementary material for: Massilia strains facilitate root growth and reshape root microbiota through diverse salicylic acid hydrolysis pathways
Source: ISME J. 2026 May 26;20(1):wrag082. doi: 10.1093/ismejo/wrag082 (PMC13215587; doi:10.1093/ismejo/wrag082)
Supplement: Supplementary_material_wrag082 [file supplementary_material_wrag082.zip › Supplementary material.docx]

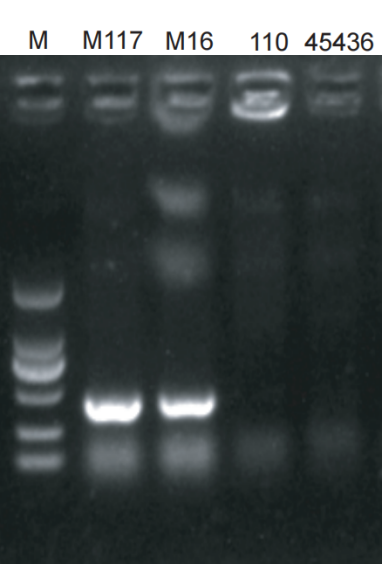


**Figure S1. Gel electrophoresis picture of four strains amplified by the *Oxaloabacteraceae*-specific primers Oxal_225f and Oxalo_656r.** Lane 1, 2, 3, 4, and 5 were marker, M117, M16, *Bradyrhizobium* USDA 110 and *Sinorhizobium* CCBAU45436, respectively.


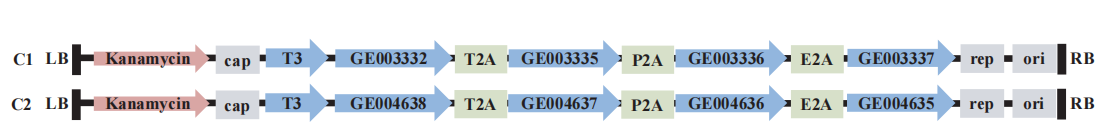


**Figure S2. Expression vector of the *NagGHAaAb* gene cluste.** The four genes were fused into a single open reading frame and are expressed under the control of a shared promoter and terminator. The individual coding sequences are linked by sequences encoding 2A peptides.


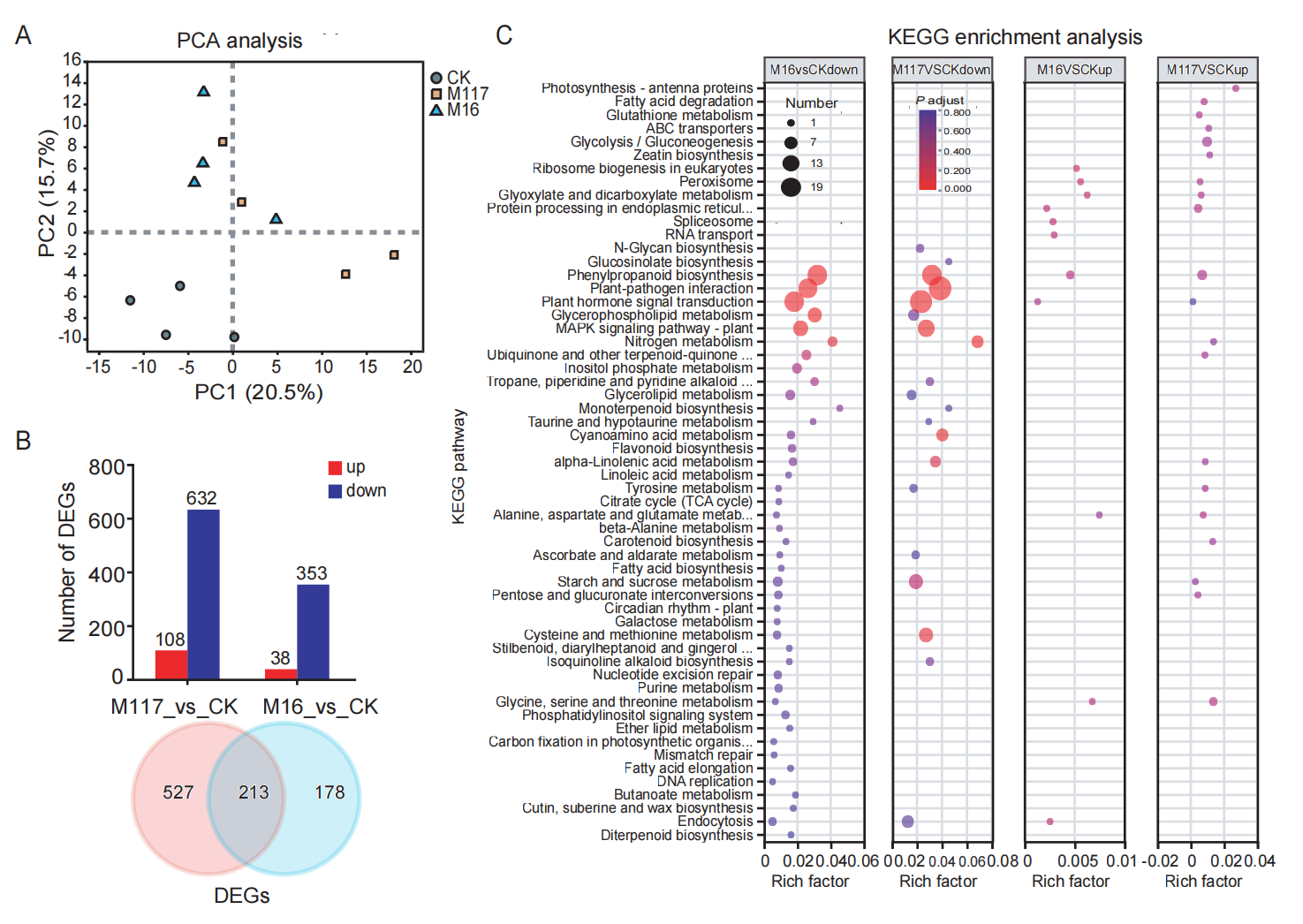


**Figure S3. Comparative transcriptomic analysis of roots of plants treated with the two *Massilia* strains.**

**A** PCA plots of transcriptome data from the roots of *Massilia-* and control-treated plants (n = 12). **B** The number of DEGs (compared with the control) in roots of plants treated with M117 or M16. **C** KEGG enrichment analysis of DEGs in root between treatment M16 or M117 and control (CK) samples.


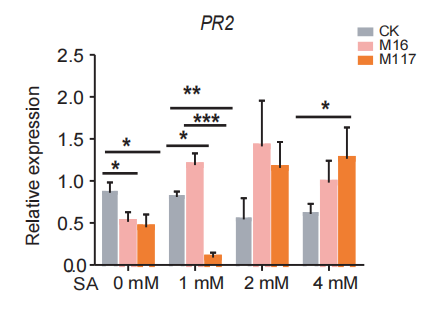


**Figure S4. Gene expression level of PR2 in control, M117- and M16-treated roots in the presence of different concentrations of SA.** Means ± SDs, n = 4 and with 3 technical replicates. Statistical analyses were performed via one-way ANOVA followed by the Newman‒Keuls test, and significance is denoted by asterisks, where * indicates P < 0.05 and *** indicates P < 0.001.


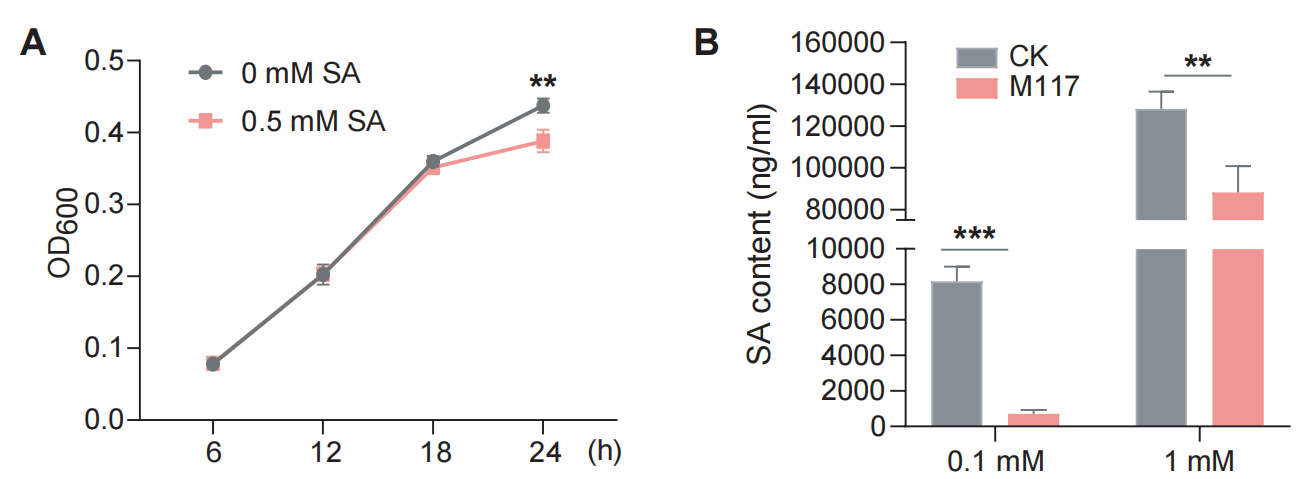


**Figure S5. Time-course analysis of M117 growth and SA degradation.**

**A** Growth curve analysis of M117 in TY with and without SA. **B** Degradation capacity of M117 for SA at varying concentrations. The values are the means ± SDs (n = 4). Statistical analyses were performed via Mann‒Whitney nonparametric tests, and significance is denoted by asterisks, where ** indicates *P* < 0.01.


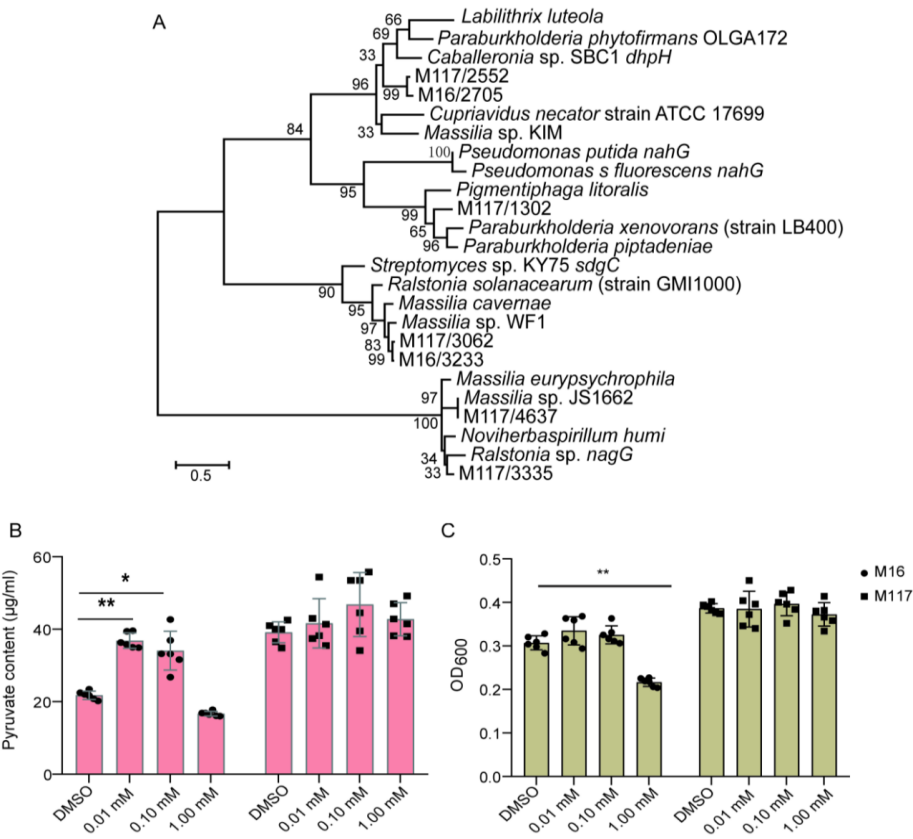


**Figure S6. Evolutionary relationship of SA hydrolysis genes and response of strains to SA.**

**A** Phylogenetic tree of SA hydroxylase genes from M117 and M16 and other species or genera built by maximum likelihood using full-length protein sequences. The branch length represents evolutionary distance. Scale bar indicates amino acid changes or substitutions per site. Pyruvate production (B) and growth (C) of M117 and M16 in MM medium under different SA concentrations. DMSO was used as a solvent control. The values are the means ± SDs (n = 4). Statistical analyses were performed via Mann‒Whitney nonparametric tests, and significance is denoted by asterisks, where ** indicates *P* < 0.01.


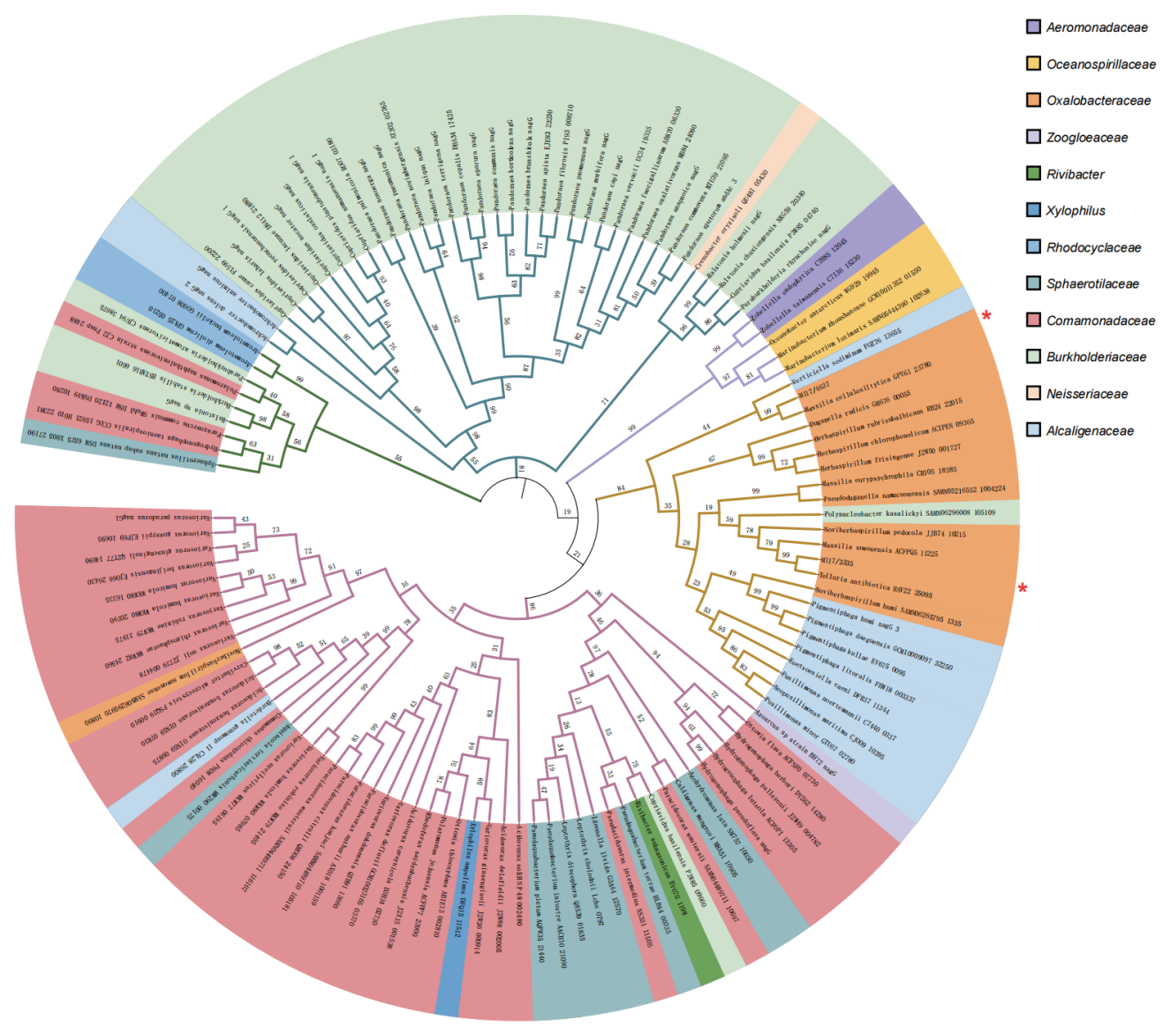


**Figure S7. Phylogenetic tree of *Massilia* *NagG* and its homologous sequences (>78% identity, from the UniProt database) based on maximum likelihood method.** Species names are color-coded according to family-level classification.


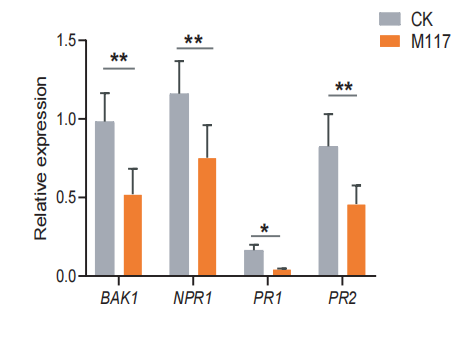


**Figure S8. Effect of M117 treatment on immune gene expression under sterile vermiculite conditions.** Mean ± SDs, n = 4 and with 3 technical replicates. Statistical analyses were performed via one-way ANOVA followed by the Newman‒Keuls test, and significance is denoted by asterisks, where * indicates P < 0.05 and ** indicates P < 0.01.


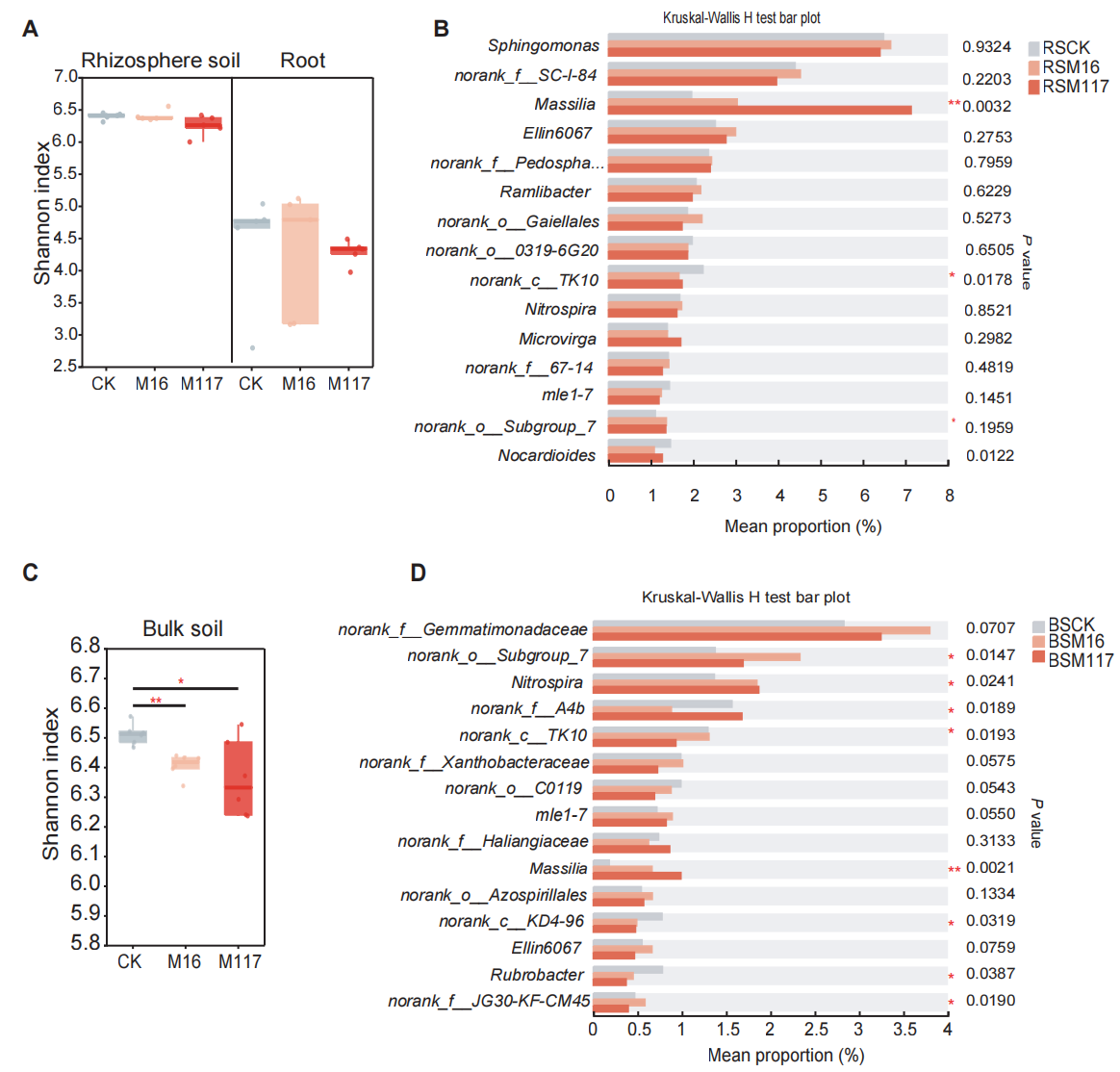


**Figure S9. The influence of *Massilia* on bacterial Shannon index and relative abundance in the different rhizocompartments**

**A** Shannon index at ASV level in root and rhizosphere soil samples across treatments. Statistical analyses were performed by the paired Wilcoxon rank-sum test. Data are presented as median values ± SDs. **B** The different relative abundances of major genera in rhizosphere soil samples among the three treatments. Significant differences between treatments were determined by the Kruskal‒Wallis H test (n=5). **C** Shannon index at ASV level in bulk soil samples across treatments. **D** The different relative abundances of major genera in bulk soil samples among the three treatments. Significant differences between treatments were determined by the Kruskal‒WallisH test (n=5).

Table S1. Specific qPCR primers designed for each gene.

| **Gene** | **Primer** | **Gene_id** | **Gene name** | **Source or**  **reference** |
| --- | --- | --- | --- | --- |
| *EDS1*-F | CACCCAAGACCCAACAAGGA | Glyma.06G187300 | Protein EDS1L | This study |
| *EDS1*-R | TGTTTGTGCTGCCCATGAGA |  |  |  |
| *TGA23*-F | CGTACGAAAGGGCTCTCCTC | Glyma.06G090900 | Transcription factor TGA23 | This study |
| *TGA23*-R | GCTCCATTATCTCGCCGTCA |  |  |  |
| *CB60G*-F | CGTTCAGGATCTCTTGCGGT | Glyma.10G148700 | Calmodulin binding protein-like | This study |
| *CB60G*-R | CGCACAAGTCTTGGCATGTT |  |  |  |
| *SARD1*_3 -F | CTGGTTGACAAAAGTGGCGG | Glyma.07G093900 | Protein SAR DEFICIENT 1 | This study |
| *SARD1*_3 -R | TTGGTGCAATCCCATCCCTC |  |  |  |
| *BAK1*-F | GGGGGCTTGCTTATTTGCAC | Glyma.05G119500 | Brassinosteroid insensitive 1-associated receptor kinase 1 | This study |
| *BAK1*-R | CGCGTACTGCGGTAGTAACA |  |  |  |
| *NPR1*-F | AGGGAGTTTTGAAGTGTGAAGT | Glyma.09G020800 | Nonexpressor of PR1 | Selig et al., 2016[49] |
| *NPR1*-R | AGGTCCAGGATCAGAGCCAT |  |  |  |
| *PR1*-F | GCAGCTAGCAAGCTACCACT | Glyma.15G06790 | Pathogenesis-related protein 1 | Selig et al., 2016[49] |
| *PR1*-R | CACGCCACAACGTTCAAGAC |  |  |  |
| *PR2*-F | GATGCACAATCCGGGGTA | Glyma.03G28850 | Pathogenesis-related protein 2 | Selig et al., 2016[49] |
| *PR2*-R | TGGCTAGATGCTAGGTTTCTG |  |  |  |

Table S2. The standard curve for SA.

| **Sample name** | **Sample type** | **Area** **(cps)** | **Is area** **(cps)** | **RT** **(min)** | **S/N** | **Target conc** | **Calculated conc.()** |
| --- | --- | --- | --- | --- | --- | --- | --- |
| STD_0.01ppb | Standard | N/A | 1.377e6 | N/A | N/A | 0.010 | N/A |
| STD_0.05ppb | Standard | N/A | 1.412e6 | N/A | N/A | 0.050 | N/A |
| STD_0.1ppb | Standard | N/A | 1.480e6 | N/A | N/A | 0.100 | N/A |
| STD_0.5ppb | Standard | 2.176e5 | 1.382e6 | 5.08 | 9.5 | 0.500 | 7.432855e-1 |
| STD_1ppb | Standard | 2.658e5 | 1.572e6 | 5.10 | 15.3 | 1.000 | 8.315918e-1 |
| STD_5ppb | Standard | 8.572e5 | 1.320e6 | 5.09 | 34.7 | 5.000 | 4.474822e0 |
| STD_10ppb | Standard | 1.912e6 | 1.530e6 | 5.08 | 59.7 | 10.000 | 9.029651e0 |
| STD_50ppb | Standard | 8.298e6 | 1.357e6 | 5.10 | 159.7 | 50.000 | 4.592134e1 |
| STD_100ppb | Standard | 1.716e7 | 1.443e6 | 5.08 | 194.4 | 100.000 | 8.972519e1 |
| STD_200ppb | Standard | 3.728e7 | 1.330e6 | 5.10 | 185.4 | 200.000 | 2.122269e2 |
| STD_500ppb | Standard | 7.349e7 | 1.106e6 | 5.10 | 213.6 | 500.000 | 5.035472e2 |

Regression Equation: y = 0.13183 x + 0.05950 (*r* = 0.999) (weighting: 1 / x)

Table S3. The standard curve for SAG.

| **Sample name** | **Sample type** | **Area** **(cps)** | **Is area** **(cps)** | **RT** **(min)** | **S/N** | **Target conc** | **Calculated conc.()** |
| --- | --- | --- | --- | --- | --- | --- | --- |
| STD_0.01ppb | Standard | 1.604e4 | 1.377e6 | 3.58 | 16.2 | 0.200 | 2.208096e-1 |
| STD_0.05ppb | Standard | 4.459e4 | 1.412e6 | 3.58 | 51.6 | 1.000 | 1.001654e0 |
| STD_0.1ppb | Standard | 6.787e4 | 1.480e6 | 3.58 | 71.4 | 2.000 | 1.561852e0 |
| STD_0.5ppb | Standard | 4.020e5 | 1.382e6 | 3.57 | 251.6 | 10.000 | 1.116489e1 |
| STD_1ppb | Standard | 7.051e5 | 1.572e6 | 3.59 | 194.9 | 20.000 | 1.734298e1 |
| STD_5ppb | Standard | 3.303e6 | 1.320e6 | 3.58 | 280.3 | 100.000 | 9.780750e1 |
| STD_10ppb | Standard | 8.607e6 | 1.530e6 | 3.57 | 247.2 | 200.000 | 2.201765e2 |
| STD_50ppb | Standard | 3.210e7 | 1.357e6 | 3.58 | 327.6 | 1000.000 | 9.264780e2 |
| STD_100ppb | Standard | 8.206e7 | 1.443e6 | 3.56 | 314.2 | 2000.000 | 2.227325e3 |
| STD_200ppb | Standard | 1.420e8 | 1.330e6 | 3.58 | 280.2 | 4000.000 | 4.184162e3 |
| STD_500ppb | Standard | 2.723e8 | 1.106e6 | 3.59 | 217.5 | 10000.000 | 9.645959e3 |

Regression Equation: y = 0.02552 x + 0.00601 (*r* = 0.998) (weighting: 1 / x)

Table S4. Primers for quantitative analysis of genes related to SA degradation.

| **Gene** | **Primer** | **Source** |
| --- | --- | --- |
| GE004637-F | GGATCTTCTACCACGGGCAC | This study |
| GE004637-R | CGGGCACACCATCTCTTTCT |  |
| GE003335-F | TGATCCAGGCCAACCTGTTC | This study |
| GE003335-R | CCAGTACTCGTACATCCCGC |  |
| GE003062-F | GGACTGTATTTCGCCCTGCT | This study |
| GE003062-R | CCCAGTGATTGAACGCTTGC |  |
| GE001302 -F | GAGCTCGTGAACTTCGTCGG | This study |
| GE001302 -R | TCATCGTCTGCACATCCTCG |  |
| GE002552-F | GACTACTCGGGCTATGTCGC | This study |
| GE002552-R | GGTACCACACCCAGTTCCAG |  |
| gene2705-F | ACTGGGTGTGGTATCGCAAG | This study |
| gene2705-R | GAACGGTTCCTGCGTTCTCT |  |
| *gyrA*-F | GCCGTACCAGGTCAACAAGA | This study |
| *gyrA*-R | GCCCGATTTATCCGACTCGT |  |

Table S5. Genomic information of 60 strains of *Massilia* species used in this study.

| **Sample name** | **Strain** | **CDS** | **rRNA** | **tRNA** |
| --- | --- | --- | --- | --- |
|  | *Massilia* sp. M117 | 6635 | 21 | 88 |
|  | *Massilia* sp. M16 | 6556 | 14 | 89 |
| GCF_002968015.1 | *Massilia* *phosphatilytica* 12-OD1 | 6283 | 6 | 73 |
| GCF_001941825.1 | *Massilia* *putida* 6NM-7 | 6604 | 21 | 87 |
| GCF_014200505.1 | *Massilia aurea* AT3.2 | 4705 | 5 | 68 |
| GCF_002752675.1 | *Massilia violaceinigra* B2 | 6474 | 17 | 87 |
| GCF_003011895.2 | *Massilia glaciei* B448-2 | 5623 | 4 | 68 |
| GCA_000740675.1 | *Massilia* sp. BSC265 | 4053 | 21 | 70 |
| GCF_011682065.1 | *Massilia rubra* CCM 8692 | 6742 | 22 | 89 |
| GCF_011682045.1 | *Massilia aquatica* CCM 8693 | 6712 | 20 | 79 |
| GCF_011682175.1 | *Massilia frigida* CCM 8695 | 6831 | 18 | 82 |
| GCF_011682145.1 | *Massilia mucilaginosa* CCM 8733 | 7005 | 23 | 75 |
| GCF_003143515.1 | *Massilia oculi* CCUG 43427 | 5214 | 22 | 82 |
| GCF_014191875.1 | *Massilia violacea* CECT 8897 | 5518 | 4 | 68 |
| GCA_900129765.1 | *Massilia* sp. CF038 | 5083 | 8 | 64 |
| GCF_007830455.1 | *Massilia lurida* CGMCC 1.10822 | 5139 | 11 | 65 |
| GCF_900116645.1 | *Massilia namucuonensis* CGMCC 1.11014 | 7275 | 16 | 68 |
| GCF_900112225.1 | *Massilia yuzhufengensis* CGMCC 1.12041 | 4970 | 10 | 67 |
| GCF_014644155.1 | *Massilia buxea* CGMCC 1.15931 | 5420 | 10 | 72 |
| GCA_021165955.1 | *Massilia* sp. DM-R-R2A-13 | 4847 | 18 | 72 |
| GCF_009857475.1 | *Massilia guangdongensis* DS3 | 5322 | 36 | 74 |
| GCF_000427785.1 | *Massilia alkalitolerans* DSM 17462 | 5491 | 7 | 63 |
| GCF_004322755.1 | *Massilia albidiflava* DSM 17472 | 6125 | 25 | 92 |
| GCF_004209755.1 | *Massilia lutea* DSM 17473 | 6258 | 25 | 88 |
| GCF_004421005.1 | *Massilia plicata* DSM 17505 | 5153 | 22 | 81 |
| GCF_000382345.1 | *Massilia niastensis* DSM 21313 | 5907 | 8 | 68 |
| GCF_009789595.1 | *Massilia flava* DSM 26639 | 5923 | 18 | 83 |
| GCF_005280315.1 | *Massilia umbonata* DSMZ 26121 | 6175 | 28 | 91 |
| GCA_014171775.1 | *Massilia* sp. Dwa41.01b | 4839 | 19 | 72 |
| GCF_008014745.1 | *Massilia arenae* GEM5 | 5201 | 12 | 74 |
| GCF_002760655.1 | *Massilia eurypsychrophila* JCM 30074 | 5484 | 13 | 69 |
| GCF_002760665.1 | *Massilia psychrophila* JCM 30813 | 4387 | 17 | 59 |
| GCF_009720745.1 | *Massilia eburnea* JCM 31587 | 5706 | 26 | 70 |
| GCA_000759615.1 | *Massilia* sp.JS1662 | 5571 | 11 | 69 |
| GCF_003590855.1 | *Massilia cavernae* K1S02-61 | 5023 | 13 | 60 |
| GCF_009720865.1 | *Massilia ginsengisoli* KCTC 42409 | 6047 | 34 | 91 |
| GCA_002007115.1 | *Massilia* sp. KIM | 4984 | 15 | 68 |
| GCA_000735635.1 | *Massilia* sp. LC238 | 5099 | 7 | 65 |
| GCA_001424165.1 | *Massilia* sp. Leaf139 | 4608 | 3 | 66 |
| GCA_015101885.1 | *Massilia* sp. LPB0304 | 4688 | 22 | 78 |
| GCF_004614185.1 | *Massilia arenosa* MC02 | 4712 | 2 | 46 |
| GCA_004798585.1 | *Massilia* sp. Mn16-1_5 | 6364 | 12 | 76 |
| GCA_009763355.1 | *Massilia* sp. NEAU-DD11 | 4908 | 23 | 68 |
| GCF_001866515.1 | *Massilia timonae* NEU | 5138 | 22 | 83 |
| GCF_004614195.1 | *Massilia horti* ONC3 | 4899 | 4 | 55 |
| GCA_900101265.1 | *Massilia* sp. PDC64 | 6516 | 15 | 77 |
| GCA_001426525.1 | *Massilia* sp. Root133 | 6067 | 4 | 73 |
| GCA_001427325.1 | *Massilia* sp. Root1485 | 6179 | 0 | 55 |
| GCA_001425685.1 | *Massilia* sp. Root335 | 5781 | 1 | 49 |
| GCA_001425045.1 | *Massilia* sp. Root351 | 6179 | 8 | 82 |
| GCA_001425265.1 | *Massilia* sp. Root418 | 6167 | 12 | 80 |
| GCF_012927275.1 | *Massilia* polaris RP-1-19 | 4219 | 6 | 64 |
| GCA_014171595.1 | *Massilia* sp. Se16.2.3 | 4820 | 19 | 72 |
| GCF_003953935.1 | *Massilia atriviolacea* SOD | 6295 | 3 | 88 |
| GCA_011682055.1 | *Massilia* sp. TW-1 | 6068 | 12 | 76 |
| GCA_001028775.2 | *Massilia* sp. WF1 | 4929 | 16 | 64 |
| GCA_001412595.2 | *Massilia* sp.WG5 | 5344 | 24 | 86 |
| GCA_003293715.1 | *Massilia* sp. YMA4 | 5315 | 22 | 82 |
| GCF_003028855.1 | *Massilia armeniaca* ZMN-3 | 5522 | 22 | 80 |
| GCF_013003915.1 | *Massilia aromaticivorans* ML15P13 | 4579 | 5 | 66 |

**Table S6. General genomic features of the two strains.**

| **General features** | **M117** | **M16** |
| --- | --- | --- |
| Accession numbers | CP186288 | CP186335 |
| Size (M) | 7.44 | 7.67 |
| Contig numbers | 1 | 1 |
| Plasmid numbers | 0 | 1 |
| Numbers of predicted genes | 6,376 | 6,556 |
| Numbers of rRNA genes | 21 | 14 |
| Numbers of tRNA genes | 94 | 89 |
| DNA G+Ccontent (mol%) | 66.03 | 65.73 |

Table S7. SA hydroxylase gene of the two strains.

| **Strain** | **Gene id** | **Gene name** | **Length（bp）** | **Swissprot annotation** | **Pathway/**  **gene** |
| --- | --- | --- | --- | --- | --- |
| M117 | GE001302 | FAD-binding protein | 1,191 | 6-hydroxynicotinate 3-monooxygenase | Ⅲ/*NahG* |
|  | GE002552 | hypothetical protein | 1,173 | Salicylate hydroxylase | Ⅲ/*NahG* |
|  | GE003062 | Bifunctional salicylyl-CoA 5-hydroxylase/oxidoreductase | 2,376 | Salicyloyl-CoA 5-hydroxylase | Ⅰ/SdgC |
|  | GE003335 | Salicylate hydroxylase | 1,266 | Salicylate 5-hydroxylase, large oxygenase component | Ⅱ/*NagGH* |
|  | GE003336 | Salicylate hydroxylase | 483 | Salicylate 5-hydroxylase, small oxygenase component |  |
|  | GE004637 | Salicylate hydroxylase | 1,263 | Salicylate 5-hydroxylase, large oxygenase component | Ⅱ/*NagGH* |
|  | GE004636 | Salicylate hydroxylase | 480 | Salicylate 5-hydroxylase, small oxygenase component |  |
| M16 | gene3233 | Bifunctional salicylyl-CoA 5-hydroxylase/oxidoreductase | 2376 | Salicyloyl-CoA 5-hydroxylase | Ⅰ/SdgC |
|  | gene2705 | FAD-dependent monooxygenase | 1,173 | Salicylate hydroxylase | Ⅲ/*NahG* |

Table S8. PERMANOVA analysis of the rhizocompartment bacterial community in different treatment based on Bray-Curtis.

| **Factors** | **Df** | **SumsOfSqs** | **MeanSqs** | **F.Model** | ***R*2** | **Pr(>F)** | **Sig.** |
| --- | --- | --- | --- | --- | --- | --- | --- |
|  |  |  | **Rhizosphere soil** |  |  |  |  |
| Treatment | 2 | 0.48052 | 0.24026 | 1.44011 | 0.19356 | 0.001 | *** |
| Residuals | 12 | 2.00201 | 0.16683 | - | 0.80644 | - |  |
| Total | 14 | 2.48253 | - | - | 1 | - |  |
|  |  |  | **Root** |  |  |  |  |
| Treatment | 2 | 2.10845 | 1.05423 | 5.95898 | 0.49828 | 0.001 | *** |
| Residuals | 12 | 2.12296 | 0.17691 | - | 0.50172 | - |  |
| Total | 14 | 4.23142 | - | - | 1 | - |  |

***, *P* < 0.001.
